# Initial Diagnosis and Detection of Very Late Local Recurrence of a Ductal Prostate Cancer due to a Ureteral Stone

**DOI:** 10.1155/2020/5392523

**Published:** 2020-02-18

**Authors:** Manolis Pratsinis, Charlotte Düwel, Olivia Köhle, Annette Enzler-Tschudy, Hans-Peter Schmid, Patrick Betschart

**Affiliations:** ^1^Department of Urology, Cantonal Hospital St. Gallen, St. Gallen, Switzerland; ^2^Department of Urology, Klinikum Rechts der Isar, Technical University of Munich, Munich, Germany; ^3^Department of Pathology, Cantonal Hospital St. Gallen, St. Gallen, Switzerland

## Abstract

We report the case of a 74-year-old patient in whom a ductal prostate cancer was incidentally endoscopically diagnosed in the course of ureteral stenting due to a left distal ureteral stone. The initial PSA was 0.8 *μ*g/l and the digital rectal examination was not suspicious. A radical prostatectomy was performed, and the ensuing follow-up was unremarkable with no signs of recurrence. Fourteen years later, the patient presented with an obstructive pyelonephritis due to a left-sided ureteral stone requiring ureteral stenting. An exophytic tumor was seen in the lining of vesicourethral anastomosis and surgically excised after the pyelonephritis subsided. The histopathological and immunohistochemical analysis revealed a ductal cancer of the prostate consistent with a late local recurrence. Serum PSA was below the limit of detection. Re-staging performed by an MRI of the pelvis, thoracoabdominal CT scan, and gallium-68 PSMA-PET did not reveal any other signs of disease. The ensuing follow-up is planned with regular flexible cystoscopy and computed thoracoabdominopelvic CT scans.

## 1. Introduction

The majority of prostate cancers are adenocarcinomas, with histological variants seen in 5–10% of carcinomas [1]. Ductal adenocarcinoma is the most common histological variant with pure ductal adenocarcinoma accounting for 0.4–0.8% of prostate cancer cases and mixed ductal adenocarcinoma seen in up to 5% of cases. The initial clinical manifestation is often hematuria and/or obstructive urinary symptoms as ductal adenocarcinomas classically arise in the central prostatic ducts [2]. Compared with acinar adenocarcinomas of the prostate, the serum prostate-specific antigen (PSA) level of ductal adenocarcinomas was seen to be 30% lower in a multivariate linear regression analysis of the SEER database performed by Morgan et al. in 2010 [3]. In the same study, ductal adenocarcinomas were also more likely high grade and had distant disease at initial presentation. Furthermore, even those patients with localized disease at diagnosis had a 2.4-fold increase of disease-specific mortality, highlighting the aggressive natural history of ductal adenocarcinomas compared with acinar adenocarcinomas [3].

In localized disease, radical prostatectomy or external beam radiation therapy is recommended. Optimal management poses a challenge for clinicians, as ductal adenocarcinomas may not express PSA, potentially delaying the diagnosis of disease and complicating follow-up.

## 2. Case Presentation

The at-the-time 60-year-old patient was referred to our clinic 14 years ago due to persistent left-sided flank pain for over six months. The patient described intermittent radiating pain to the left groin and denied micturition disorders such as urinary discomfort or gross hematuria. Left-sided hydronephrosis was seen on renal ultrasound, and a kidney, ureter, bladder (KUB) plain film radiography was performed demonstrating an 8 mm large opaque structure in projection of the distal ureter, suspicious for a ureteral stone as well as a 10 mm large bladder stone. Stone therapy with transurethral lithotripsy and ureteral stenting on the left was planned. During cystoscopy, an exulcerating lesion with whitish, fibrinous necrotic slough was seen left of verumontanum, with no other lesions apparent. The bladder stone was disintegrated and removed using a grasping forceps, and a biopsy of the tumor was performed. The ureteral stone was pushed back into the left renal pelvis and a double-pigtail stent was inserted. Further stone treatment was performed by extracorporeal shock wave lithotripsy with removal of the ureteral stent after radiological disintegration of the stone was seen.

The histopathological analysis revealed a ductal adenocarcinoma of the prostate. Digital rectal examination of the prostate was not suspicious, and total serum PSA was 0.8 *μ*g/l. The tumor markers carcinoembryonic antigen (CEA), carbohydrate antigen 19-9 (CA 19-9), and cancer antigen 125 (CA 125) were normal. Staging was conducted with a pelvic MRI, thoracoabdominal CT scan, and bone scintigraphy without evidence of metastasis. A radical prostatectomy with extended pelvic lymphadenectomy was performed, with ductal adenocarcinoma seen in both prostate lobes without extraprostatic extension, nodal metastasis, or positive surgical margins, corresponding to a pT2c, pN0, cM0, Gleason score 7b (4 + 3), R0 stage. The postoperative course was uneventful, and the patient was subjected to regular clinical follow-up with digital-rectal examination and serum PSA control. Serum PSA was not detectable in the first postoperative control and remained below the limit of detection throughout the whole duration of follow-up. The patient had persistent postoperative stress urinary incontinence, which required the use of 1-2 pads per day with an otherwise unremarkable follow-up.

Fourteen years after the initial diagnosis, the now 74-year-old patient was admitted to the emergency department with fever and left-sided flank pain. A computed tomography of the abdomen revealed an 8 mm stone in the distal left ureter with consecutive dilation of the left ureter and perirenal stranding of the left kidney indicating obstructive pyelonephritis. Intravenous antibiotic therapy was initiated, and the immediate ureteral stenting of the left ureter was performed with a double-pigtail stent. During cystoscopy, a solitary 15 mm large tumorous lesion was seen on the left side of vesicourethral anastomosis, which was not resected due to the urosepsis. The patient quickly recovered from the infection and was subjected to a transurethral resection of the tumor and semirigid ureteroscopy for stone removal. The tumorous lesion of the bladder was resected in toto and the ureterolithiasis successfully removed.

**Figure 1:**
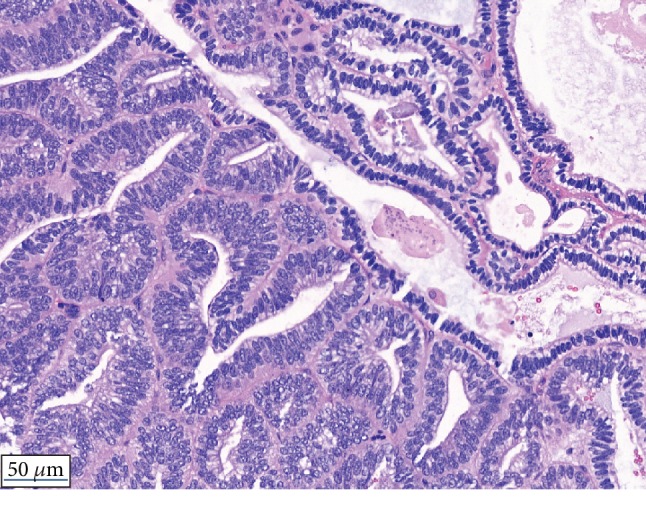
Hematoxylin and eosin staining. Ductal adenocarcinoma of the prostate with pseudoendometrioid growth pattern, focally even reminiscent of secreting endometrium.

**Figure 2:**
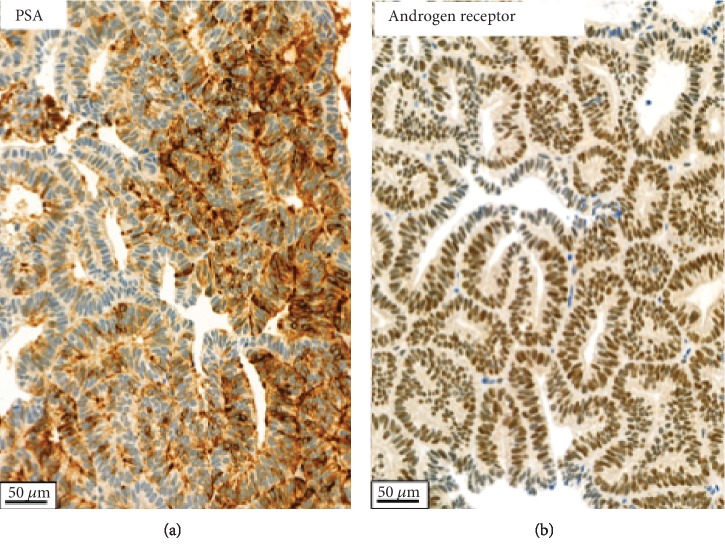
Immunohistochemical staining. Ductal adenocarcinoma of the prostate with focal expression of PSA (a). Nuclear expression of androgen receptor in approximately 60% of tumor cells (b).

The histological analysis revealed a ductal adenocarcinoma consistent with a local recurrence of the known ductal adenocarcinoma of the prostate (Figure 1). Immunohistochemical analysis revealed a low focal PSA positivity and androgen receptor (AR) positivity in approximately 60% of tumor cells (Figure 2). The Ki67 proliferation index was 3%.

Imaging with a pelvic MRI, thoracoabdominal CT scan, and a gallium-68 PSMA-PET scan revealed no signs of metastasis. The case was discussed at our multidisciplinary tumor board, and external beam radiation therapy of the prostate bed was recommended to reduce the risk of recurrence. The patient wished to abstain from further therapy so as not to worsen the preexisting stress urinary incontinence. A flexible cystoscopy was performed 3 months postoperatively demonstrating an unremarkable vesicourethral anastomosis. Further follow-up is scheduled with annual cystoscopies and thoracoabdominopelvic CT scans.

## 3. Discussion

In the reported case, a very late local recurrence was incidentally detected fourteen years after initial diagnosis. Prior to that, regular follow-up had been performed with digital rectal examinations and serum PSA detection, with no signs of local recurrence and a serum PSA which was below detection level. Furthermore, the Ki67 proliferation index of the resected local recurrence was very low at 3%, indicating a very slowly growing disease, which may partially explain the long latency period between primary diagnosis and recurrence.

As with the initial diagnosis, the recurrence was only detected due to left-sided ureterolithiasis. Chung et al. performed a retrospective population-based case-control study to assess the association of prostate cancer and urinary calculi. While they were able to show that the incidence of prior urinary calculi was higher in patients with newly diagnosed prostate cancer compared with the control group (21% vs. 14.1%), no significant relationship was seen in regard to ureter calculi [4].

The treatment of localized disease of ductal adenocarcinomas of the prostate consists of radical prostatectomy or external beam radiation therapy. When compared with acinar adenocarcinomas of the prostate, ductal cancers were more likely to present with advanced disease and a lower PSA indicating the challenging diagnosis [3]. Patients who were subjected to surgical treatment by radical prostatectomy appeared to have a higher risk of extraprostatic extension, positive surgical margins [5, 6], and a higher local recurrence rate [7] when compared with acinar adenocarcinomas of the prostate. Nonetheless, there is currently no recommended specific alterations of follow-up compared with acinar adenocarcinomas of the prostate in the latest prostate cancer guidelines of the European Association of Urology [8]. A recent retrospective analysis of twenty-seven patients treated with radiotherapy and androgen deprivation therapy by Bergamin et al. recommended including regular CT scans in follow-up as local recurrences and metastasis occurred at undetectable PSA levels [9], but prospective data regarding the optimal follow-up is lacking.

## 4. Conclusion

Ductal adenocarcinomas of the prostate are rare histological variants and should be treated as a separate tumor entity with an individualized management. When localized disease is present, a radical prostatectomy or external beam radiation therapy can be performed. The further follow-up has to be tailored to the individual patient, as PSA is not a reliable tool for assessing the existence of disease. Furthermore, regular thoracoabdominopelvic imaging, for instance, with regular CT or MRI scans as well as flexible cystoscopies should be considered.

## References

[1] Epstein J. I., Cubilla A. L., Humphrey P. A. (2011). Tumors of the prostate gland, seminal vesicles, penis, and scrotum. *AFIP Atlas of Tumor Pathology Series*.

[2] Hertel J. D., Humphrey P. A. (2011). Ductal adenocarcinoma of the prostate. *The Journal of Urology*.

[3] Morgan T. M., Welty C. J., Vakar-Lopez F., Lin D. W., Wright J. L. (2010). Ductal adenocarcinoma of the prostate: increased mortality risk and decreased serum prostate specific antigen. *The Journal of Urology*.

[4] Chung S. D., Liu S. P., Lin H. C. (2013). Association between prostate cancer and urinary calculi: a population-based study. *PLoS One*.

[5] Samaratunga H., Duffy D., Yaxley J., Delahunt B. (2010). Any proportion of ductal adenocarcinoma in radical prostatectomy specimens predicts extraprostatic extension. *Human Pathology*.

[6] Brinker D. A., Potter S. R., Epstein J. I. (1999). Ductal adenocarcinoma of the prostate diagnosed on needle biopsy: correlation with clinical and radical prostatectomy findings and progression. *The American Journal of Surgical Pathology*.

[7] Tu S. M., Lopez A., Leibovici D. (2009). Ductal adenocarcinoma of the prostate: clinical features and implications after local therapy. *Cancer*.

[8] Mottet N., van den Bergh R. C. N., Briers E. (2019). EAU – ESTRO – ESUR – SIOG guidelines on prostate cancer. https://uroweb.org/wp-content/uploads/EAU-ESUR-ESTRO-SIOG-Guidelines-on-Prostate-Cancer-large-text-V2.pdf.

[9] Bergamin S., Eade T., Kneebone A. (2019). Ductal carcinoma of the prostate: an uncommon entity with atypical behaviour. *Clinical Oncology*.

